# Latest Advancements in Next-Generation Semiconductors: Materials and Devices for Wide Bandgap and 2D Semiconductors

**DOI:** 10.3390/mi14111992

**Published:** 2023-10-27

**Authors:** Zeheng Wang, Jing-Kai Huang

**Affiliations:** 1 Manufacturing, CSIRO, Lindfield, NSW 2070, Australia; 2 Department of Systems Engineering, City University of Hong Kong, Kowloon, Hong Kong; 3 School of Materials Science and Engineering, University of New South Wales (UNSW), Sydney, NSW 2052, Australia

Semiconductor materials, devices, and systems have become indispensable pillars supporting the modern world, deeply ingrained in various facets of our daily lives [1,2]. In the realm of computing, semiconductors serve as the backbone of microprocessors and memory units, enabling the rapid calculations and data storage that power everything from personal computers to large-scale data centers [3,4,5]. When it comes to power conversion, semiconductor-based components like transistors and diodes are critical in optimizing energy efficiency, whether in renewable energy systems or electric vehicles [6,7,8,9]. Moreover, in the field of optoelectronics, semiconductor technologies have revolutionized lighting and detecting solutions through the development of energy-efficient and long-lasting light-emitting diode (LED) technology [10,11,12,13], which has not only transformed the way we illuminate our surroundings, but also enhanced the precision and sensitivity of various detection and sensing applications [14,15,16]. Moreover, in the ever-expanding domain of information processing, semiconductors are at the heart of information storage, communication devices, and complex integrated circuits that facilitate the seamless exchange and long-duration storage of data [17,18].

The Special Issue on “Latest Advancements in Next-Generation Semiconductors: Materials and Devices for Wide Bandgap and 2D Semiconductors” serves as a comprehensive repository of cutting-edge research that extends the frontiers of semiconductor devices, optoelectronics, and material science. The articles in this issue delve into a myriad of challenges and opportunities that characterize these rapidly evolving disciplines. Specifically, the contributions are organized around four pivotal themes.

## 1. Device Architecture and Design

The field of semiconductor devices has experienced significant advancements, particularly in the design and performance of high-electron-mobility transistors (HEMTs) [19,20]. For instance, the paper by Meng Zhang et al. explores the influence of gate geometry on AlGaN/GaN nanochannel HEMTs, revealing that tri-gate designs offer higher peak transconductance and frequency performance compared to dual-gate structures. Another study by Haiwu Xie et al. delves into the irradiation effects on tunnel field-effect transistors, offering optimization strategies for better reliability against irradiation.

This Special Issue also covers innovative device designs and the challenges associated with crucial environments. A study by Choi and colleagues on AlGaN/GaN HEMTs with HfO2 as the passivation layer shows improvements in breakdown voltage, but highlights the need for balancing this with frequency characteristics. Similarly, research on quartz flexible accelerometers by Zhigang Zhang et al. discusses the suppression of noise through high-pass filtering.

## 2. Material Science and Fabrication Techniques

Material science and fabrication engineering play crucial roles in the performance and reliability of electronic devices [21,22,23]. Han et al. investigate the impact of oxygen flow rates on the electrical characteristics of amorphous indium–gallium–zinc oxide transistors, emphasizing the importance of controlling oxygen vacancies. In another study, Kudryashov and colleagues explore the use of ultrashort laser pulses for inscribing photoluminescent micro-bits inside dielectric crystals, opening new avenues for optomechanical memory storage. These two developments indicate that using proper material science and fabrication engineering innovations can still further boost the performance of semiconductor components [24,25].

## 3. Energy Efficiency and Power Management

Energy efficiency and power management are other areas that have received considerable attention in semiconductor-based systems. For example, a study by Weizhong Chen et al. on FIN-LDMOS with a bulk electron accumulation effect shows promising results in terms of specific on-resistance, which may be beneficial for high-power telecommunication applications [26]. Another paper by Boyang Ma et al. focuses on enhancing the ESD performance of power-rail clamp circuits, offering a design that is strengthened against false triggers [27]. In addition, power management is a critical aspect of modern electronic systems. One paper by Dan Butnicu in this Special Issue focuses on the derating-sensitive failure rate of tantalum polymer capacitors within DC-DC eGaN-FET-based PoL converters, providing insights into improving the reliability of these systems [28].

## 4. Optoelectronics and Light-Based Technologies

Lastly, this issue features comprehensive reviews that offer a broader perspective on this field. One such review by Zhaoyong Liu et al. focuses on the advancements in integrating meta-surface structures with micro-LEDs, providing a roadmap for future research [15].

In summary, the articles in this Special Issue collectively contribute to the advancement of micro- and nanoelectronics, offering innovative solutions and highlighting areas that require further investigation. As the guest editors, it is our privilege to present this collection of pioneering research that will undoubtedly serve as a valuable resource for scholars and industry professionals alike.
